# Determination of plant silicon content with near infrared reflectance spectroscopy

**DOI:** 10.3389/fpls.2014.00496

**Published:** 2014-09-24

**Authors:** Adriaan Smis, Francisco Javier Ancin Murguzur, Eric Struyf, Eeva M. Soininen, Juan G. Herranz Jusdado, Patrick Meire, Kari Anne Bråthen

**Affiliations:** ^1^Ecosystem Management Research Group, University of AntwerpAntwerp, Belgium; ^2^Department of Arctic and Marine Biology, UiT - The Arctic University of NorwayTromsø, Norway

**Keywords:** NIRS, plant silica concentration, calibration, Fennoscandia, ecosystem research, graminoids, *Deschampsia cespitosa*

## Abstract

Silicon (Si) is one of the most common elements in the earth bedrock, and its continental cycle is strongly biologically controlled. Yet, research on the biogeochemical cycle of Si in ecosystems is hampered by the time and cost associated with the currently used chemical analysis methods. Here, we assessed the suitability of Near Infrared Reflectance Spectroscopy (NIRS) for measuring Si content in plant tissues. NIR spectra depend on the characteristics of the present bonds between H and N, C and O, which can be calibrated against concentrations of various compounds. Because Si in plants always occurs as hydrated condensates of orthosilicic acid (Si(OH)_4_), linked to organic biomolecules, we hypothesized that NIRS is suitable for measuring Si content in plants across a range of plant species. We based our testing on 442 samples of 29 plant species belonging to a range of growth forms. We calibrated the NIRS method against a well-established plant Si analysis method by using partial least-squares regression. Si concentrations ranged from detection limit (0.24 ppmSi) to 7.8% Si on dry weight and were well predicted by NIRS. The model fit with validation data was good across all plant species (*n* = 141, *R*^2^ = 0.90, RMSEP = 0.24), but improved when only graminoids were modeled (*n* = 66, *R*^2^ = 0.95, RMSEP = 0.10). A species specific model for the grass *Deschampsia cespitosa* showed even slightly better results than the model for all graminoids (*n* = 16, *R*^2^ = 0.93, RMSEP = 0.015). We show for the first time that NIRS is applicable for determining plant Si concentration across a range of plant species and growth forms, and represents a time- and cost-effective alternative to the chemical Si analysis methods. As NIRS can be applied concurrently to a range of plant organic constituents, it opens up unprecedented research possibilities for studying interrelations between Si and other plant compounds in vegetation, and for addressing the role of Si in ecosystems across a range of Si research domains.

## Introduction

Silicon (Si) is widely present in the plant kingdom. In most plant species, plant Si constitutes ~0.1 up to 2% of Si by plant dry weights (Epstein, 1994; Hodson et al., 2005). Remarkably high average concentrations in some plant groups such as horsetails (~3%Si by dry weight), and grasses (~1.5%Si by dry weight; Hodson et al., 2005) suggest that Si can have an essential role in plants. Si supports plant growth, improves the plant structural strength, viability, reproduction and resistance against biotic (e.g., parasitism, pests, herbivory) and abiotic (e.g., metal toxicity, salinity, drought) stresses, as well as increases the efficiency of light interception (Epstein, 1999; Ma et al., 2001). Hence there are compelling reasons to study Si in plants. However, the importance of plant Si reaches beyond the plant kingdom as the Si-assimilation of plants is coupled to the global biogeochemical Si and C cycling (Street-Perrott and Barker, 2008). Plants significantly increase mineral silicate weathering and the coupled CO_2_ drawdown (Kelly et al., 1998; Moulton et al., 2000). Plant Si uptake functions as a “terrestrial Si filter” (Struyf and Conley, 2012), controlling the continuous delivery of Si to rivers that connect the continental Si cycle to the coastal and oceanic Si cycle (Tréguer and De La Rocha, 2013). As such, plant biosilicification is tightly linked to aquatic eutrophication problems (Cloern, 2001), and the strength of the oceanic carbon sequestration (Ragueneau et al., 2006), making the study of plant Si in terrestrial ecosystems instrumental to climate change studies.

Despite the increasing interest, the quantification of biogenic silica (bSi) in terrestrial ecosystems is hampered by the time-intensive and relatively expensive chemical analysis methods for bSi. In most studies, the total plant Si content, further indicated as plant bSi, is analyzed by chemical digestion followed by a colorimetric or a spectrophotometric analysis method (e.g., Massey et al., 2007; Saccone et al., 2007; Vandevenne et al., 2012; Carey and Fulweiler, 2013; Meunier et al., 2013), or by wet or dry ashing followed by gravimetric analysis (e.g., Carnelli et al., 2001; Ma et al., 2001; Blecker et al., 2006). These methods are destructive, time-intensive and require chemical pre-treatment. Especially in large-scale studies and monitoring programs, more efficient methods are warranted for a better understanding of both the role of Si in ecosystems as well as the biosilicification at the scale of entire ecosystems.

In this study, we address the applicability of a comparable non-destructive method, Near Infrared Reflectance Spectroscopy (NIRS), for the analysis of Si in plants. NIRS has been used intensively in agricultural and industrial research (e.g., Shenk and Westerhaus, 1994), and is receiving increasing interest in ecology after numerous successful ecological applications have been demonstrated, mostly for the determination of plant primary and secondary organic compounds and parameters related to the food quality of plants (e.g., Stolter et al., 2006; Chodak, 2008). Plant material absorbs near infrared radiation (NIR) where there is presence of chemical bonds between hydrogen (H) and other atoms (e.g., C–H, N–H, O–H; Kaye, 1954). Si occurs in plants in and between plant cells as amorphous opal silica structures called phytoliths or phytogenic Si (Sangster and Hodson, 1986; Piperno, 1988). These structures are always composed of hydrated condensates of orthosilicic acid (Si(OH)_4_), containing mostly Si-O-Si bonds, and some hydroxyl groups (SiO_2_.nH_2_O; Currie and Perry, 2007). In addition, these silica structures show strong interactions with several plant biomolecules (e.g., cellulose; Perry and Lu, 1992). Although Si never directly binds to H, the hydrated silica structures probably interact with their plant organic environment through hydroxyl groups (Zhang et al., 2013) and are expected to result in a specific NIR absorption signature of plant bSi. This suggests a possibility to apply NIRS as analysis method of plant Si.

The reasons to apply NIRS are manifold: samples need minor pre-treatment without the use of chemicals, the analysis itself is rapid, non-destructive, allows repeated measurements, and it provides information about many plant parameters from a single sample (Foley et al., 1998). Recently, X-ray fluorescence (XRF) spectroscopy was presented as a more rapid and accurate alternative to digestion-based chemical techniques (Reidinger et al., 2012). Although XRF spectroscopy has shown to have the same advantages regarding analysis time, analysis cost, and sample consumption, XRF spectroscopy cannot be used for the simultaneous analysis of numerous plant organic compounds for which NIRS have been shown to be successful and which are often of great interest in ecological research (e.g., Schaller et al., 2012).

The applicability of NIRS for Si analysis has been tested using agricultural (alfalfa hay, rice straw) and bio-industrial products (sugar mill mud and ash; Halgerson et al., 2004; Jin and Chen, 2007; Ostatek-Boczynski et al., 2013). Although they all suggested a potential applicability of NIRS, none of these studies showed sufficiently strong calibrations for direct application on plant material. Jin and Chen (2007) used the insoluble ash method for their reference values, although this method was only recommended if the content of indigestible mineral residues is sought (Van Soest and Jones, 1968). Halgerson et al. (2004) used a calibration data set with a very narrow concentration range around and average Si concentration of 0.02%, and Ostatek-Boczynski et al. (2013) only tested NIRS on industrially processed plant material (sugar mill by-products). For ecological applications, the widespread nature of Si in the plant kingdom calls for testing the general applicability of NIRS, i.e., a method that can be applied both to the wide range of plant taxa and over the typical concentration range in which bSi is found in nature. With support from these moderately successful pioneer studies, we hypothesize in this study that NIRS may be applied for measuring the Si content of plants.

## Materials and methods

### Plant sampling

The study was performed in the alpine parts of the northern, subarctic zone of Norway (Finnmark). Plant species were sampled from eight river valleys, extending the temperature gradient of the region. The sampled plant species (Table 1) are typical for the riparian zone, dominated by willows, forbs and grasses, and for the surrounding dry heath tundra, dominated by dwarf shrubs. Each sample consisted of several plant individuals from the same location, and habitat, with a maximum distance of 25 m from each other. Plant individuals were always sampled by harvesting the green non-woody plant parts; the whole plant in the case of grasses, forbs and horsetails, and the leaves in the case of shrubs (Table 1). Plant material was cut some centimeters above the soil level and sampled plant material was always visually controlled to be free from soil particles. Samples of 29 plant species, representing different growth forms, were collected making a total of 442 plant samples (Table 1). In order to cover plant and Si structural variations over several plant development stages, we sampled plant species at the beginning (June), during (July) and at the end of the growing season (August).

**Table 1:** **Overview of plant species, plant part, sample size (*n*), and the plant silicon concentration range, average value and the coefficient of variation of the plant silicon concentration for all studied growth forms with species alphabetically ordered within each growth form**.

**Growth form *Plant species***	**Plant part**	**Total**	**Silicon concentration (% on dry weight)**		
			**Range (min–max)**	**Average value**	**Coefficient of variation (%)**
***Graminoids***		**265**	**0.072–9.991**	**1.054**	**195**
* Agrostis tenuis*	WP	7	0.396–3.868	1.351	92
* Alopecurus pratensis*	WP	2	0.201–0.778	0.489	83
* Anthoxanthum nipponicum*	WP	21	0.177–1.287	0.541	45
* Avenella flexuosa*	WP	56	0.084–1.066	0.419	54
* Calamagrostis sp*.	WP	23	0.290–3.060	1.417	48
* Carex bigelowii*	WP	35	0.073–2.912	0.690	86
* Deschampsia cespitosa*	WP	75	0.341–9.991	1.575	71
* Festuca ovina*	WP	3	0.424–1.142	0.728	51
* Nardus stricta*	WP	30	0.555–3.620	1.728	35
* Phleum alpinum*	WP	13	0.114–0.411	0.250	37
***Forbs***		**99**	**0.002–2.875**	**0.203**	**232**
* Alchemilla sp*.	WP	40	0.003–2.875	0.454	147
* Bistorta vivipara*	WP	11	0.002–0.021	0.009	71
* Chamaepericlenum suecicum*	WP	2	0.007–0.007	0.007	4
* Comarum palustre*	WP	1	–	0.068	–
* Geranium sylvaticum*	WP	9	0.003–0.025	0.012	58
* Ranunculus sp*.	WP	4	0.036–0.634	0.196	149
* Rubus chamaemorus*	WP	2	0.056–0.072	0.064	18
* Rumex acetosa*	WP	7	0.009–0.037	0.019	57
* Solidago virgaurea*	WP	10	0.018–0.143	0.058	83
* Trientalis europaea*	WP	1	–	0.006	–
* Trollius europaeus*	WP	6	0.002–0.034	0.016	75
* Viola sp*.	WP	6	0.006–0.040	0.022	62
***Shrubs and Dwarf shrubs***		**50**	**0.000–0.053**	**0.010**	**100**
* Betula nana/pubescens*	L	5	0.000–0.010	0.004	86
* Empetrum nigrum*	L	12	0.003–0.053	0.015	87
* Salix sp*.	L	28	0.001–0.039	0.011	86
* Vaccinium myrtillus*	L	4	0.003–0.011	0.006	59
* Vaccinium vitis-ideae*	L	1	–	0.001	–
***Horsetails***		**26**	**0.038–7.797**	**2.909**	**71**
* Equisetum sp*.	WP	26	0.038–7.797	2.909	71

*WP, whole plant; L, leaves*.

### The reference chemical analysis method

Plant samples were oven dried for 3 days at 75°C. We chose not to wash the plant samples as sampling routines excluded the presence of soil particles and the ecological context (high precipitation, dense vegetation, long distance to roads) restricted the possibilities for aeolean dust/soil transport. Subsequently, samples were pulverized using a ball mill (Mixer Mill, MM301; Retsch GmbH and Co. Haan, Germany) in order to get very fine and homogeneous plant material. We applied a chemical digestion followed by a colorimetric analysis, as the gravimetric approach has been proven to show a poor reproducibility (Herbauts et al., 1994). Plant bSi was extracted by the wet alkaline (0.1 M Na_2_CO_3_) extraction, a technique recently confirmed to be suitable for bSi analysis of plant material (Meunier et al., 2013). About 30 mg of pulverized plant material was incubated for 4 h in 0.1 M Na_2_CO_3_ at 80°C (DeMaster, 1991) and then 10 ml of the extract was filtered (Chromafil® A-45/25, pore size of 0.45 μm). After extraction, samples were stored in a dark room at 3°C and were analyzed colorimetrically for extracted dissolved Si (DSi) within a maximum of 2 weeks, using a Continuous Flow Analyser (CFA; SKALAR SA 1500, Smith and Milne, 1981). Blank extractions were subtracted to account for Si release from recipients and chemicals. The error of the entire analysis procedure was measured as the (reproducibility) standard deviation of the analysis of 12 subsamples of a single pulverized plant sample (*Deschampsia cespitosa*).

### Analysis with NIRS

Remaining pulverized plant samples were made into tablets (Ø16 mm, >1 mm thick) by applying 6 tons of pressure with a hydraulic press, in order to obtain a homogeneous flat surface. A homogeneous flat surface reduces random light scatter of loose powder, thus reducing random variation in spectral signatures. Before NIRS analyses, the plant tablets were oven dried at 50°C for 2 h in order to remove the remaining water film on the hydrophilic plant material. The dry samples were then cooled in a desiccator until scanning by NIRS. The NIRS-spectra, measured as the logarithm of the inversed reflectance (log(1/R)), were recorded with monochromatic radiation at 1.4 nm intervals from 350 to 1050 nm, and at 2 nm intervals from 1000 to 2500 nm, using a FieldSpec 3 (ASD Inc., Boulder, Colorado, USA) and were registered at 1 nm interpolated intervals. Each sample spectrum was recorded as the average of three replicate scans. NIRS scanning was performed at room temperature during one single winter day, which most likely resulted in a stable room temperature as there was no sunlight to warm up the room.

### Calibration and validation

Three nested calibration sets were created; one on the total sample size, representing all studied plant species (*n* = 442); one for a subset containing all graminoids (*n* = 264), which are, as active Si accumulators, most often studied in plant-Si studies, and finally, in order to test the performance of NIRS when applied to one single plant species, one calibration for a subset containing all samples of the grass species *Deschampsia cespitosa* (*n* = 75). All data sets were treated similarly: in order to get a similar spectral diversity within the calibration and validation dataset, calibration and validation samples were selected using the Kennard-Stone algorithm (Kennard and Stone, 1969) included in the “prospectr” package 0.1.3 (Stevens and Ramirez-Lopez, 2014) in R 3.0.1 (R Development Core Team, 2013). Spectral outliers were identified by means of Mahalanobis distances (75% quantile as cutoff point; Mahalanobis, 1936; De Maesschalck et al., 2000) and removed in a single operation. Before starting the calibration, the wavelength regions where the NIRS sensors overlapped (350–380 nm, 760–840 nm, 1700–1800 nm and 2450–2500 nm) were removed to avoid false correlations from instrumental noise.

The statistical procedure for the calibration, using partial least-squares regression (Martens and Næs, 1993), in the pls-package 2.4–3 (Mevik et al., 2013) in R 3.1.0. Different transformations on the spectral data set were considered for the calibrations: scaling and/or centering, smoothing before applying derivatives, and the use of first (1D) and second order derivatives (2D) by using the earlier mentioned “prospectr” package (Stevens and Ramirez-Lopez, 2014) in R.

The most parsimonious calibration model was chosen based on evaluation of two calibration statistics: a high coefficient of multiple determination (*R*^2^), and the lowest root mean square error of calibration (RMSEC) for a given number of selected model components *k*. Overfitting was avoided by selecting the combination of model parameters *k* showing the lowest standard error of cross-validation (SECV; Mark and Workman, 1991). Finally, the chosen calibration model was used to predict the Si concentrations of the validation sample sets. The *R*^2^, the RMSEP (“root mean square error of prediction,” i.e., the mean error rate between predicted and real values), the bias (i.e., the systematic error of the linear regression), and the intercept and slope of the linear fit of the predictions were used to assess the applicability of the developed NIRS calibration models. We tested the quality of all calibration models with and without the visible region (380–720 nm). Chlorophyll has a strong signature in the visible light spectra, and seasonal variation in chlorophyll content could consequently be confounded with variation in bSi content. As the models with visible light were consistently of lower quality, we only present models without the visible light.

## Results

### Plant Si concentrations

**Figure 1:**
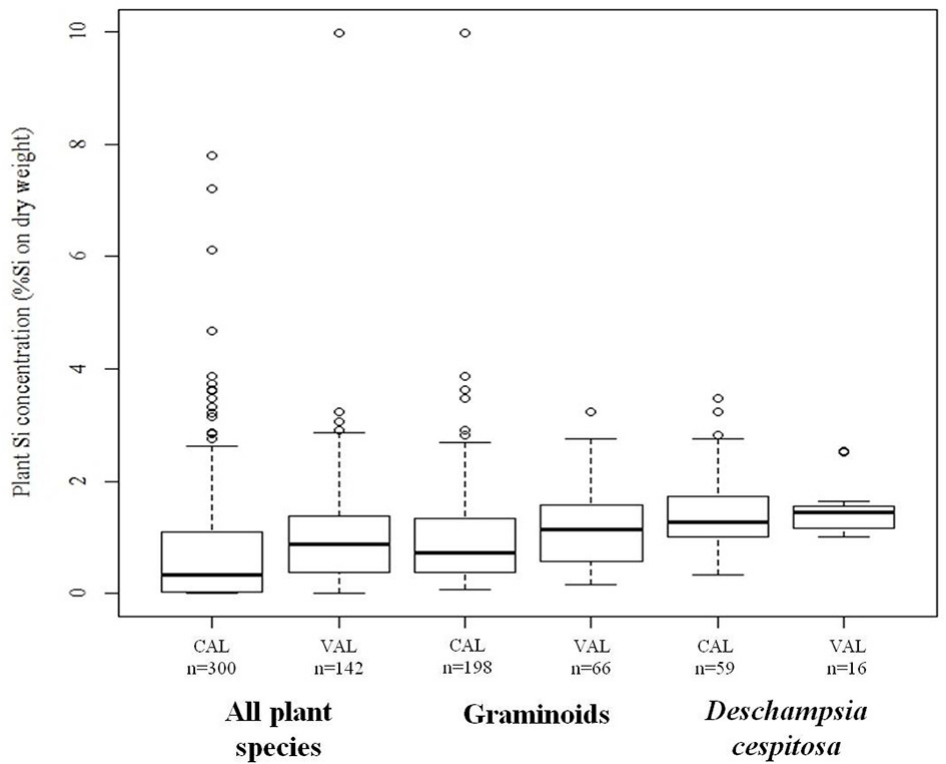
**Statistical summary (minimum, first quartile, median, third quartile, maximum value which is not an outlier, and outliers) of the plant silicon (Si) concentrations, measured by chemical analysis, in the calibration (CAL) and validation (VAL) dataset of the “all species” model, the “graminoids” model, and the “*Deschampsia cespitosa*” model**.

We found plant Si concentrations to range from the detection limit (<0.024%Si) to 10.0% of Si by dried plant weight. The average error of the wet chemical analysis method, measured as the reproducibility standard deviation of the repeated measurement of a *Deschampsia cespitosa* sample with average concentration of 0.34%Si, was 0.022% of Si by dry weight, which corresponds to an average relative error of 2.6%. Across all species, plant Si concentration showed large variation with a coefficient of variation of 69%. However, the concentrations of Si were low (<0.1%Si) for almost all shrubs, dwarf shrubs and forbs, while most graminoids and horsetails showed average Si concentrations higher than 0.5% (Table 1). The selection of the calibration and validation datasets based on the spectral characteristics of the samples resulted in comparable Si concentration frequency distributions of the calibration and validation datasets for the three separate models (Figure 1).

### The NIRS calibration models

**Figure 2:**
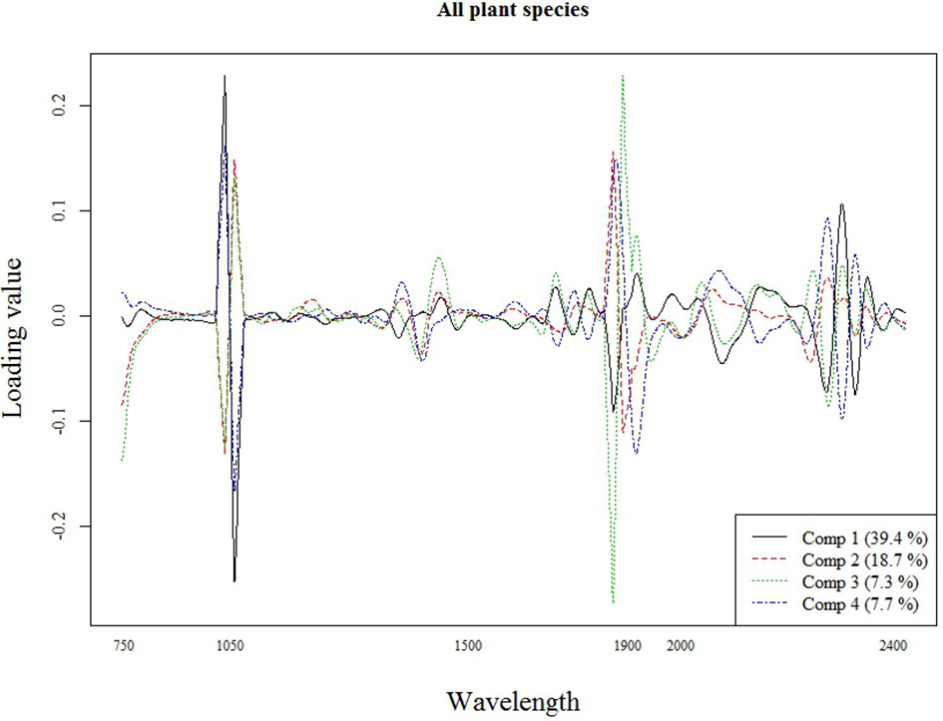
**Loading plot for the most important components of the calibration model for all plant species**.

**Figure 3:**
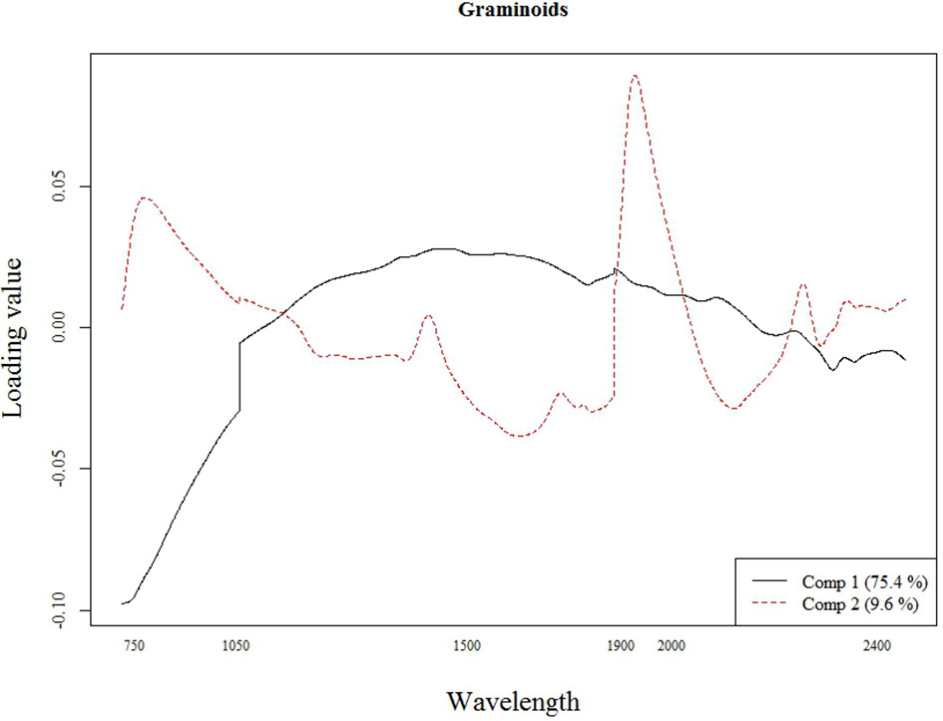
**Loading plot for the most important components of the calibration model for the graminoids**.

**Figure 4:**
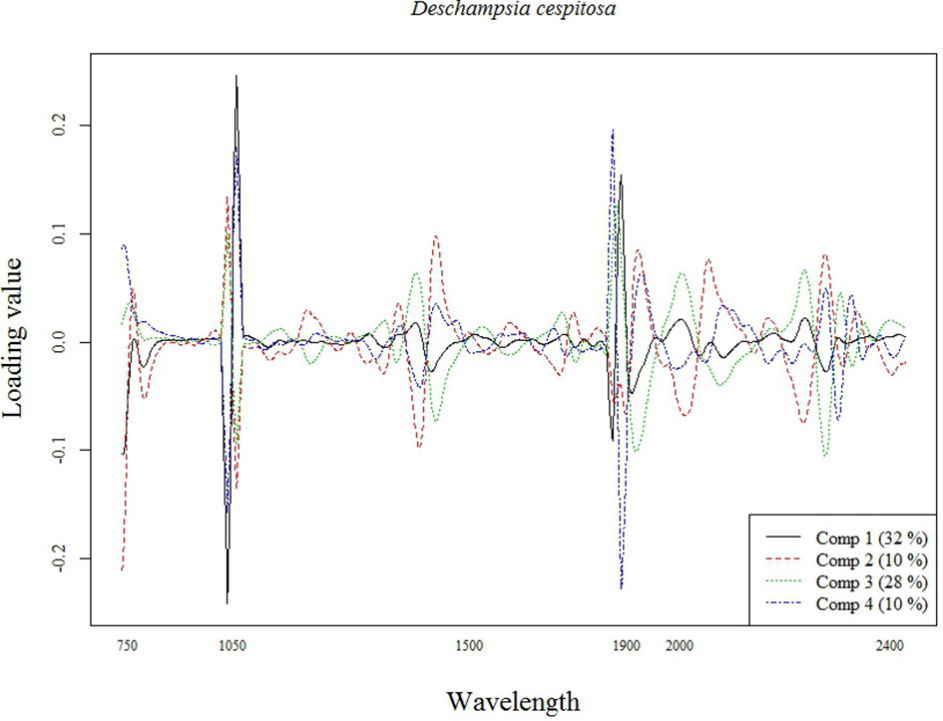
**Loading plot for the most important components of the calibration model for *Deschampsia cespitosa***.

The best calibration model for Si across all plant species was based on the second order derivatives (2D) of the smoothed (17 wavelength intervals) NIR spectra with 23 components, which showed highest loading values at 1050, 1900, and 2300 nm (Figure 2). The best calibration model for graminoids was based on the multiplicative-scatter corrected spectra, and 33 components. Highest loading values were found for wavelengths around 1900 and 2300 nm (Figure 3). Finally, the best calibration model for the grass species *Deschampsia cespitosa* was based on the centered, second order derivative spectra with 19 components, with highest loading values for the same wavelengths as the calibration model including all plant species (Figure 4).

**Figure 5:**
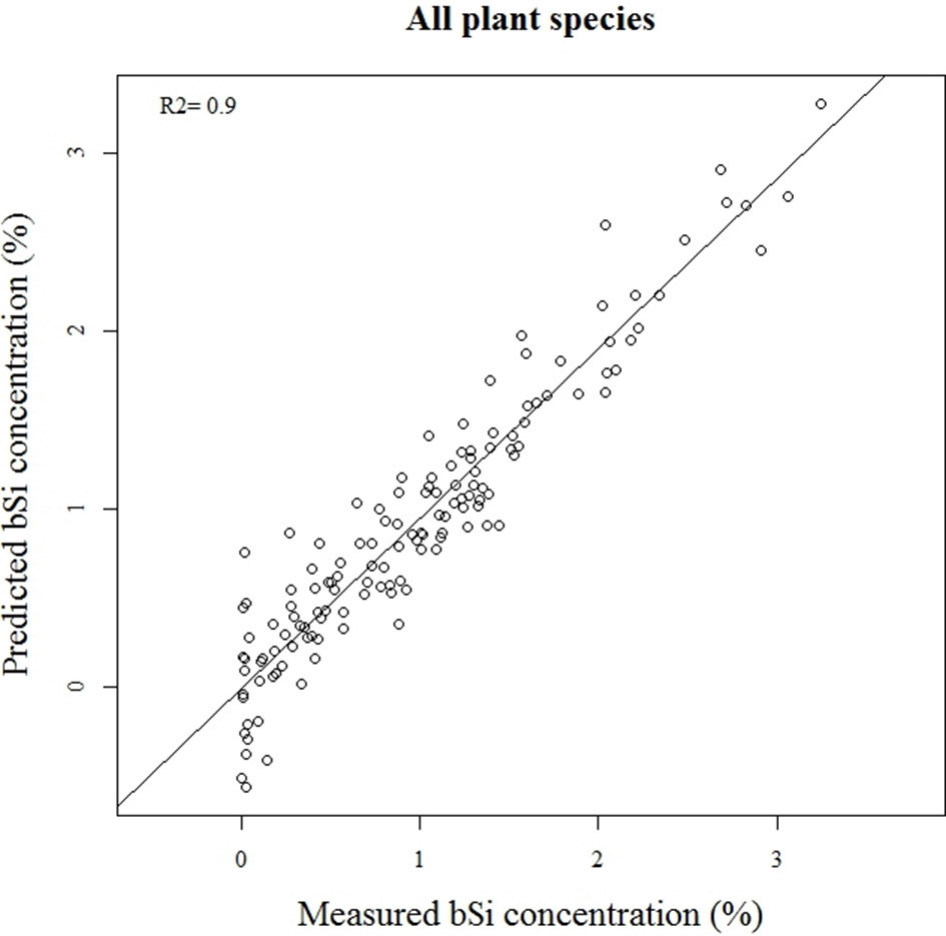
**Validation plot of the NIRS calibration model based on samples of all the plant species**.

**Figure 6:**
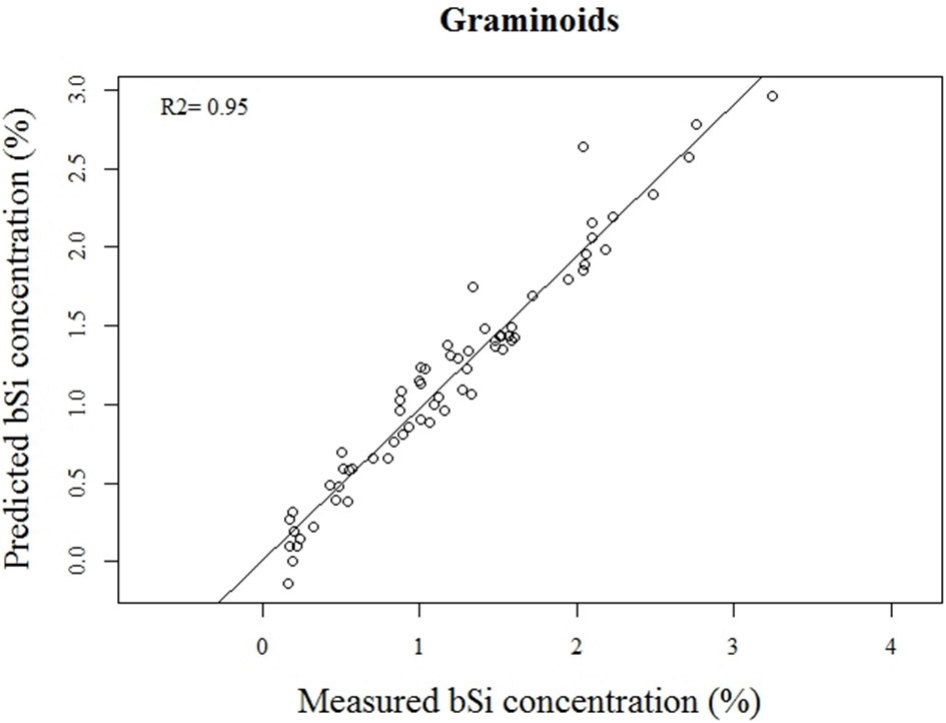
**Validation plot of the NIRS calibration model based on samples of graminoids**.

**Figure 7:**
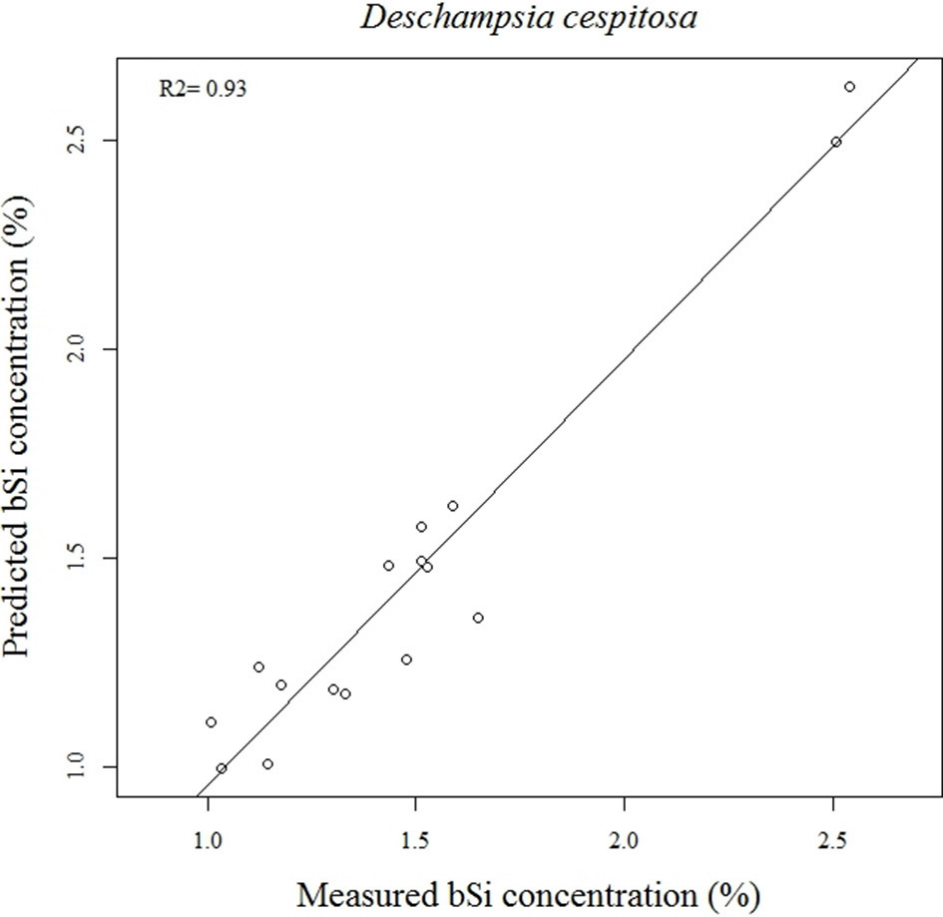
**Validation plot of the NIRS calibration model based on samples of *Deschampsia cespitosa***.

Our calibration model including 442 samples from 29 different plant species showed a RMSEP of 0.24% of Si by dry weight (Table 2, Figure 5), corresponding to a relative error of 28% for the average measured Si concentration. Our separate calibration model for graminoids showed a RMSEP of 0.10% of Si by dry weight, corresponding to an average relative error of only 8.3% (Table 2, Figure 6). Narrowing the graminoid dataset to the most abundant grass species, *Deschampsia cespitosa*, increased the calibration model accuracy; a RMSEP of 0.023% of Si by dry weight, corresponding to a relative error of 1.3% (Table 2, Figure 7). The bias was also highest for the model including all plant species, whereas it was lowest and similar for the graminoids and *Deschampsia cespitosa* (Table 2). For the models including all plant species and the graminoids, the slope and intercept of the validation regression lines were close to 0 and 1 respectively, whereas the *Deschampsia cespitosa* model showed a slope close to one, and an intercept which differed more from 0 (Table 2). The *R*^2^ values of the validation regression were all equal to or higher than 0.90 and showed a similar pattern as the bias, with the highest value for the graminoids model (0.95), the lowest for the model including all plant species (0.90) and an intermediate value for the *Deschampsia cespitosa* model (0.93; Table 2, Figures 5–7).

**Table 2:** **Calibration and validation statistics (*R*^2^: coefficient of determination; RMSEC: root mean square error of calibration; RMSEP: root mean square error of prediction) of the Partial Least Squares regression models on NIR spectra for the analysis of plant Si concentration in three separate models**.

**Calibration model**	**Model selection: calibration**				**Model quality: validation**					
	**No. of components (k)**	***n***	***R*^2^**	**RMSEC**	***n***	***R*^2^**	**RMSEP**	**Bias**	**Intercept**	**Slope**
All plant species	23	300	0.88	0.3327	141	0.90	0.2379	−0.054	−0.0009	0.95
Graminoids	33	198	0.91	0.2076	66	0.95	0.1021	−0.032	0.0070	0.97
*Deschampsia cespitosa*	19	59	0.95	0.1357	16	0.93	0.0150	−0.035	−0.0660	1.02

## Discussion

### The quality of the NIRS calibration model

Our results show that the Si content of plants can successfully be measured by NIRS across a large range of plant species. All three calibration models showed a high coefficient of determination (*R*^2^ ≥ 0.9). The calibration model for the grass *Deschampsia cespitosa* showed the best overall performance with the lowest root mean square error in the validation (RMSEP), and a slope closest to 1 (Table 2). Taking into account that the average error of the wet chemical extraction method followed by a colorimetric analysis is 0.022% of Si by dry weight, using NIRS for graminoids increases the average error with 0.078% of Si by dry weight, which is acceptable when working with plants accumulating Si (Si > 0.5%Si by dry weight; Raven, 2003). For the *Deschampsia cespitosa* model, the average error became slightly lower compared to the reference chemical analysis method (0.015%Si vs. 0.022%Si). Also, a further specification of the calibration model from all plant species to the plant group level and finally to a single plant species increased the accuracy. Prudence is called for when using the calibration model across all plant species in studies that require very high accuracy. Although the model containing all plant species showed a high *R*^2^, and an intercept and slope close to 0 and 1, respectively, it was characterized by a RMSEP of 0.24% of Si by dry weight, which is rather high, even for plant species actively accumulating Si.

Our three calibration models showed remarkably better validation statistics compared to earlier studies on the application of NIRS of plant material (Halgerson et al., 2004; Jin and Chen, 2007; Ostatek-Boczynski et al., 2013), and is probably a result of (1) the use of a well-proven and widely used reference method for plant Si analysis (Meunier et al., 2013), and (2) a calibration dataset which consists of plant species representing almost the entire range of natural plant Si concentrations (Hodson et al., 2005). We also hypothesize that the specific pre-treatment of the plant samples before NIRS scanning, where we pressed ground plant powder into a tablet, may have increased the quality of the calibration. The ball-mill produced very fine powder, resulting in a flat tablet surface which may have reduced both light scattering from powder and original structural differences between plant species. However, as we were not able to measure the fineness, particle size distribution and homogeneity of the ground plant samples, this issue remains subject for further research and the quality of the NIRS calibration could potentially be increased by using a cyclotech mill instead of the ball mill procedure. The cyclotech mill grinding method ensures a narrow particle size distribution, which has been proven to strongly influence the NIR spectrum (Casler and Shenk, 1985).

### NIRS calibration and plant Si biochemistry

Our success in measuring Si with NIRS across several plant lineages, representing different plant phenological stages and a range of ecological contexts, may also be due to the single type of Si structure in plants. Whereas the often transient and species-specific character of associations between minerals and organic or hydrated molecules (Clark et al., 1987) may limit the applicability of NIRS for mineral analysis to a well-defined population (plant species, sampling time; Foley et al., 1998), Si in plants is only known to occur as a hydrated condensate of orthosilicic acid (Si(OH)_4_). However, narrowing the calibration dataset to graminoids and finally to *Deschampsia cespitosa* resulted in a clear increase in accuracy (lower RMSEP) and a regression slope close to one in the case of *Deschampsia cespitosa*. We suggest that this increase in calibration model accuracy is related to plant group/species dependent locations of bSi formation. Cell-silicification shows clear phylogenetic differences. In monocotyledons, silica is more often deposited in the cell lumen of “silica cells,” and as “silica bodies” on cells, while dicotyledons more often show silica deposits within or underneath the cell wall (Piperno, 1988). In addition, plant lineages differ in the type of tissues which are silicified; in grasses, Si is preferentially deposited as a layer beneath the cuticle (Prychid et al., 2004), the Pteridaceae (ferns) and Equisetaceae (horsetails) mostly show silicification within and at epidermal cells (Kaufman et al., 1971; Sundue, 2009), while orchids (Orchidaceae) are characterized by silica bodies in the sclerenchyma (Møller and Rasmussen, 1984). Moreover, depending on the condensation environment (e.g., Si(OH)_4_ concentration, temperature, pH), the silica condensate can differ significantly in density and composition (Perry et al., 2003). Si also shows strong interactions with plant biomolecules such as cellulose (Perry and Lu, 1992), phenol- or lignin-carbohydrate complexes (Inanaga and Okasaka, 1995; Inanaga et al., 1995), callose (Law and Exley, 2011), and proteins (Perry and Keeling-Tucker, 2003). Although little is known about these chemical interactions, Si possibly links to these complex plant biomolecules through dihydrogen bonds (Zhang et al., 2013). As different condensation locations are characterized by different plant biomolecules, this strong interaction with the organic plant environment may result in an additional chemical and spectral difference in silica between plant lineages, which may explain the better calibration model for graminoids separately. Plant lineages show a large variation in the absolute bSi concentration (Hodson et al., 2005), possibly also influencing the characteristics of the individual Si condensates and its interactions with the organic environment. Finally, the three calibration models may also be confounded by other plant compounds as lignin, cellulose, and phenolics, which are, in some plant families, closely related to plant silica (Schoelynck et al., 2010; Cooke and Leishman, 2012). However, our dataset was not large enough to compare NIRS calibration model characteristics for different plant groups and further research is needed to assess the importance of plant group/species specific calibrations.

### Method efficiency and cost, and future challenges

Using NIRS for plant Si analysis significantly reduces the operational and time costs. The time for sample drying is dependent on the amount of samples dried at the same time, but in all cases it is small and identical for both methods, and is therefore omitted from this estimation. In both methods, it took about 5 min to grind each sample using a ball-mill. In the reference method, about 30 mg of each plant sample was weighed, bSi was extracted in an alkaline solution and the extract was analyzed spectrophotometrically. Because this reference method was already highly optimized, e.g., by using an automated spectrophotometric device (SKALAR, 332 samples per run), each of these three steps only took about 3 min. When plant Si was analyzed by using NIRS scanning, it took 3 min per sample for the production of a tablet and 1.5 min per sample for the NIRS scan itself. As such, our calibration shows that the use of NIRS may reduce the analysis time by at least 30% compared to the chosen reference method for plant Si analysis, which is comparable to that reported for XRF spectroscopy (Reidinger et al., 2012). However, most time was still spent on the pre-treatment (drying, grinding, making tablet) of the plant sample. We believe that this pre-treatment may explain our strong calibration across largely differing plant species. However, omitting the grinding and rather extending the NIRS method to dried unground plant samples may be worthwhile as it results in a considerable progress in time efficiency, reducing the analysis time to 1.5 min after drying (90% reduction). The application to unground samples would introduce the structure of the plant leaf as an important calibration factor. Leaf structure strongly differs between plant species. Although a scatter correction of the NIR spectra may reduce the influence of structure (e.g., Gislum et al., 2004), the earlier described plant family specific location of silica deposition probably requires separate calibrations for each plant family or even plant species. Application of NIRS on fresh plant material, which would exclude all sample pre-treatment and allow for *in situ* non-destructive field monitoring of plant silicon, shows some larger challenges as all wavelength zones with high influence on our calibration model were situated in zones typical for O–H bonds of water, which should be avoided when working with fresh plant samples (e.g., Foley et al., 1998; Morón et al., 2007). Finally, we recommend other studies to carefully consider whether washing of the plant samples is needed in order to remove dust/soil particles. This will extend the analysis time, but is a common factor for both the chemical and the spectral analysis method.

An even stronger gain in efficiency and reduction in operational costs is obtained when applying NIRS for simultaneous analysis of different plant compounds. As a result of its central role in the functioning of plants, Si biochemistry in plants is closely linked to that of numerous other chemical compounds (Cooke and Leishman, 2011). Studies that quantify Si in plants often also quantify plant structural compounds such as lignin and cellulose (Schoelynck et al., 2010), as well as compounds related to abiotic and biotic plant stress (toxic metals, secondary metabolites) and plant macronutrients (e.g., N and P). Numerous studies have shown the applicability of NIRS for the analysis of many of these Si-related plant compounds (e.g., Joffre et al., 1992; Gillon et al., 1999; Stolter et al., 2006; González-Martín et al., 2007). Especially in studies where focus is on different plant compounds, the advantages of using NIRS as a method are unprecedented both in efficiency and cost.

## Conclusions

Our study shows that NIRS can be used to analyze silicon in plants, and as such represents a new promising method for measuring Si more effectively, at least 30% faster, and at minimal operational cost. We showed the applicability of a calibration model based on plant species across different plant families, and that accuracy can be gained by applying plant group or species-specific calibration models. Although XRF spectroscopy offers comparable advantages, we regard XRF and NIRS to be complementary with regards to the possibility of simultaneously analyzing elementary and organic compounds, respectively. As such, both methods provide scope for studies characterizing variation of bSi in natural ecosystems at much larger scales than previously possible with traditional chemical digestion methods and will open avenues for doing research on the role of Si in plants and ecosystems.

## Author contributions

Kari Anne Bråthen developed the research hypothesis and the study design. Adriaan Smis performed field sampling and chemical analysis, Francisco Javier Ancin Murguzur performed NIR scanning, calibration model development, and statistical analysis. The final manuscript is the end product of joint writing efforts of all authors.

### Conflict of interest statement

The authors declare that the research was conducted in the absence of any commercial or financial relationships that could be construed as a potential conflict of interest.

## References

[B1] Blecker S. W. McCulley R. L. Chadwick O. A. Kelly E. F. (2006). Biologic cycling of silica across a grassland bioclimosequence. Glob. Biogeochem. Cycles 20, GB3023 10.1029/2006GB002690

[B2] Carey J. C. Fulweiler R. W. (2013). Nitrogen enrichment increases net silica accumulation in a temperate salt marsh. Limnol. Oceanogr. 58, 99–111 10.4319/lo.2013.58.1.0099

[B3] Carnelli A. L. Madella M. Theurillat J.-P. (2001). Biogenic silica production in selected alpine plant species and plant communities. Ann. Bot. 87, 425–434 10.1006/anbo.2000.1355

[B4] Casler M. D. Shenk J. S. (1985). Effect of sample grinding size on forage quality estimates of smooth bromegrass clones. Crop Sci. 25, 167–170 10.2135/cropsci1985.0011183X002500010040x

[B5] Chodak M. (2008). Application of near infrared spectroscopy for analysis of soils, litter and plant materials. Pol. J. Environ. Stud. 17, 631–642

[B6] Clark D. H. Mayland H. F. Lamb R. C. (1987). Mineral analysis of forages with near infrared reflectance spectroscopy. Agron. J. 79, 485–490 10.2134/agronj1987.00021962007900030016x

[B7] Cloern J. E. (2001). Our evolving conceptual model of the coastal eutrophication problem. Mar. Ecol. Prog. Ser. 210, 223–253 10.3354/meps210223

[B8] Cooke J. Leishman M. R. (2011). Is plant ecology more siliceous than we realise? Trends Plant Sci. 16, 61–68 10.1016/j.tplants.2010.10.003 21087891

[B8a] Cooke J. Leishman M. R. (2012). Tradeoffs between foliar silicon and carbon-based defences: evidence from vegetation communities of contrasting soil types. Oikos 121, 2052–2060 10.1111/j.1600-0706.2012.20057.x

[B9] Currie H. A. Perry C. C. (2007). Silica in plants: biological, biochemical and chemical studies. Ann. Bot. 100, 1383–1389 10.1093/aob/mcm247 17921489 PMC2759229

[B10] De Maesschalck R. Jouan-Rimbaud D. Massart D. L. (2000). The Mahalanobis distance. Chemometr. Intell. Lab. 50, 1–18 10.1016/S0169-7439(99)00047-7

[B11] DeMaster D. J. (1991). Measuring biogenic silica in marine sediments and suspended matter. Geophys. Monogr. 63, 363–367

[B12] Epstein E. (1994). The anomaly of silicon in plant biology. Proc. Natl. Acad. Sci. U.S.A. 91, 11–17 10.1073/pnas.91.1.11 11607449 PMC42876

[B13] Epstein E. (1999). Silicon. Annu. Rev. Plant Phys. 50, 641–664 10.1146/annurev.arplant.50.1.641 15012222

[B14] Foley W. J. McIlwee A. Lawler I. Aragones L. Woolnough A. P. Berding N. (1998). Ecological applications of near infrared reflectance spectroscopy – a tool for rapid, cost-effective prediction of the composition of plant and animal tissues and aspects of animal performance. Oecologia 116, 293–305 10.1007/s004420050591 28308060

[B15] Gillon D. Houssard C. Joffre R. (1999). Using near-infrared reflectance spectroscopy to predict carbon, nitrogen and phosphorus content in heterogeneous plant material. Oecologia 118, 173–182 10.1007/s004420050716 28307692

[B16] Gislum R. Micklander E. Nielsen J. P. (2004). Quantification of nitrogen concentration in perennial ryegrass and red fescue using near-infrared reflectance spectroscopy (NIRS) and chemometrics. Field Crop. Res. 88, 269–277 10.1016/j.fcr.2004.01.021

[B17] González-Martín I. Hernández-Hierro J. M. González-Cabrera J. M. (2007). Use of NIRS technology with a remote reflectance fiber-optic probe for predicting mineral composition (Ca, K, P, Fe, Mn, Na, Zn), protein and moisture in alfalfa. Anal. Bioanal. Chem. 387, 2199–2205 10.1007/s00216-006-1039-4 17205269

[B18] Halgerson J. L. Sheaffer C. C. Martin N. P. Peterson P. R. Weston S. J. (2004). Near-infrared reflectance spectroscopy prediction of leaf and mineral concentrations in Alfalfa. Agron. J. 96, 344–351 10.2134/agronj2004.0344

[B19] Herbauts J. Dehalu F. A. Gruber W. (1994). Quantitative determination of plant opal content in soils, using a combined method of heavy liquid separation and alkali dissolution. Eur. J. Soil Sci. 45, 379–385 10.1111/j.1365-2389.1994.tb00522.x

[B20] Hodson M. J. White P. J. Mead A. Broadley M. R. (2005). Phylogenetic variation in the silicon composition of plants. Ann. Bot. 96, 1027–1046 10.1093/aob/mci255 16176944 PMC4247092

[B21] Inanaga S. Okasaka A. (1995). Calcium and silicon binding compounds in cell walls of rice shoots. Soil Sci. Plant Nutr. 41, 103–110 10.1080/00380768.1995.10419563

[B22] Inanaga S. Okasaka A. Tanaka S. (1995). Does silicon exist in association with organic compounds in rice plants? Soil Sci. Plant Nutr. 41, 111–117 10.1080/00380768.1995.10419564

[B23] Jin S. Chen H. (2007). Near-infrared analysis of the chemical composition of rice straw. Ind. Crop. Prod. 26, 207–211 10.1016/j.indcrop.2007.03.00421174169

[B24] Joffre R. Gillon D. Dardenne P. Agneesens R. Biston R. (1992). The use of near-infrared spectroscopy in litter decomposition studies. Ann. Sci. For. 49, 481–488 10.1051/forest:1992050418306785

[B25] Kaufman P. B. Bigelow W. C. Schmid R. Ghosheh N. S. (1971). Electron microprobe analysis of silica of epidermal cells in Equisetum. Am. J. Bot. 58, 309–316 10.2307/2441411

[B26] Kaye W. (1954). Near infrared spectroscopy - I. Spectral identification and analytical application. Spectrochim. Acta 6, 257–287 10.1016/0371-1951(54)80011-7

[B27] Kelly E. F. Chadwick O. A. Hilinski T. E. (1998). The effect of plants on mineral weathering. Biogeochemistry 42, 21–53 10.1023/A:1005919306687

[B28] Kennard R. W. Stone L. A. (1969). Computer aided design of experiments. Technometrics 11, 137–148 10.1080/00401706.1969.10490666

[B29] Law C. Exley C. (2011). New insight into silica deposition in horsetail (Equisetum arvense). Plant Biol. 11:112 10.1186/1471-2229-11-112 21801378 PMC3160890

[B30] Ma J. F. Miyake Y. Takahashi E. (2001). Silicon as a beneficial element for crop plants, in Silicon in Agriculture. Studies in Plant Science, Vol. 8, eds Datnoff L. E. Snyder G. H. Korndörfer G. H. (Amsterdam: Elsevier), 17–39

[B31] Mahalanobis P. C. (1936). On the generalised distance in statistics. Proc. Nat. Instit. Sci. India 12, 49–55

[B32] Mark H. Workman J. (1991). Statistics in Spectroscopy. New York, NY: Academic

[B33] Martens H. Næs T. (1993). Multivariate Calibration. New York, NY: Wiley

[B34] Massey F. P. Ennos A. R. Hartley S. E. (2007). Grasses and the resource availability hypothesis: the importance of silica-based defences. J. Ecol. 95, 414–424 10.1111/j.1365-2745.2007.01223.x

[B35] Meunier J. D. Keller C. Guntzer F. Riotte J. Braun J. J. Anuparna K. (2013). Assessment of the 1% Na_2_CO_3_ technique to quantify the phytolith pool. Geoderma 216, 30–35 10.1016/j.geoderma.2013.10.014

[B36] Mevik B.-H. Wehrens R. Liland K. H. (2013). Package ‘pls’: Partial Least Squares and Principal Component regression in R. Available online at: http://cran.r-project.org/web/packages/pls/pls.pdf

[B37] Møller J. D. Rasmussen H. (1984). Stegmata in Orchidales: character state distribution and polarity. Bot. J. Linn. Soc. 89, 53–76 10.1111/j.1095-8339.1984.tb01000.x

[B38] Morón A. Garcia A. Sawchik J. Cozzolino D. (2007). Preliminary study of the use of near-infrared reflectance spectroscopy to assess nitrogen content of undried wheat plants. J. Sci. Food Agric. 87, 147–152 10.1002/jsfa.2691

[B39] Moulton K. L. West J. Berner R. A. (2000). Solute flux and mineral mass balance approaches to the quantification of plant effects on silicate weathering. Am. J. Sci. 300, 539–570 10.2475/ajs.300.7.539

[B40] Ostatek-Boczynski Z. A. Purcell D. E. Keeff E. C. Martens W. N. O'Shea M. G. (2013). Rapid determination of carbon, nitrogen, silicon, phosphorus, and potassium in sugar mill by-products, mill mud, and ash using near infrared spectroscopy. Commun. Soil Sci. Plan. 44, 1156–1166 10.1080/00103624.2012.756004

[B41] Perry C. C. Belton D. Shafran K. (2003). Studies of biosilicas; structural aspects, chemical principles, model studies and the future. Prog. Mol. Subcell. Biol. 33, 269–299 10.1007/978-3-642-55486-5_11 14518377

[B42] Perry C. C. Keeling-Tucker T. (2003). Model studies of colloidal silica precipitation using biosilica extracts from Equisetum telmatia. Colloid Polym. Sci. 281, 652–664 10.1007/s00396-002-0816-7

[B43] Perry C. C. Lu Y. (1992). Preparation of silicas from silicon complexes: role of cellulose in polymerisation and aggregation control. J. Chem. Soc. Faraday Trans. 88, 2915–2921 10.1039/ft9928802915

[B44] Piperno D. R. (1988). Phytolith Analysis: An Archaeological and Geological Perspective. San Diego: Academic 10.1126/science.241.4873.1694-a 17820900

[B45] Prychid C. J. Rudall P. J. Gregory M. (2004). Systematics and biology of silica bodies in monocotyledons. Bot. Rev. 69, 377–440 10.1663/0006-8101(2004)069[0377:SABOSB]2.0.CO;2

[B46] Ragueneau O. Schultes S. Bidle K. Claquin P. Moriceau B. (2006). Si and C interactions in the world ocean: importance of ecological processes and implications for the role of diatoms in the biological pump. Glob. Biogeochem. Cycles 20, GB4S02 10.1029/2006G.B.002688

[B47] Raven J. A. (2003). Cycling silicon – the role of accumulation in plants. New Phytol. 158, 419–430 10.1046/j.1469-8137.2003.00778.x 36056512

[B48] R Development Core Team (2013). R: A language and Environment for Statistical Computing. Vienna: R Foundation for Statistical Computing. Available online at: http://www.R-project.org

[B49] Reidinger S. Ramsey M. H. Hartley S. E. (2012). Rapid and accurate analyses of silicon and phosphorus in plants using a portable X-ray fluorescence spectrometer. New Phytol. 195, 699–706 10.1111/j.1469-8137.2012.04179.x 22671981

[B50] Saccone L. Conley D. J. Sauer D. (2007). Methodologies for amorphous silica analysis. J. Geochem. Exp. 88, 235–238 10.1016/j.gexplo.2005.08.045

[B51] Sangster A. G. Hodson M. J. (1986). Silica in higher plants, in Silicon Biochemistry, eds Evered D. O'Connor M. (Chichester: John Wiley), 90–107

[B52] Schaller J. Brackhage C. Dudel G. E. (2012). Silicon availability changes structural carbon ratio and phenol content of grasses. Environ. Exp. Bot. 77, 283–287 10.1016/j.envexpbot.2011.12.009

[B53] Schoelynck J. Bal K. Backx H. Okruszko T. Meire P. Struyf E. (2010). Silica uptake in aquatic and wetland macrophytes: a strategic choice between silica, lignin and cellulose? New Phytol. 186, 385–391 10.1111/j.1469-8137.2009.03176.x 20136720

[B54] Shenk J. S. Westerhaus M. O. (1994). The application of near infrared spectroscopy (NIRS) to forage analysis, in National Conference on Forage Quality Evaluation and Utilization, eds Fahey G. C. Jr. Mosser L. E. Mertens D. R. Collins M. (Madison: American Society of Agronomy), 406–449

[B55] Smith J. D. Milne P. J. (1981). Spectrophotometric determination of silicate in natural waters by formation of α-molybdosilicic acid and reduction with a tin(IV)-ascorbic acid-oxalic acid mixture. Anal. Chim. Acta 123, 263–270 10.1016/S0003-2670(01)83179-2

[B56] Stevens A. Ramirez-Lopez L. (2014). An Introduction to the “Prospectr” Package in R. Available online at: http://cran.r-project.org/web/packages/prospectr/prospectr.pdf

[B57] Stolter C. Julkunen-Tiitto R. Ganzhorn J. U. (2006). Application of near infared reflectance spectroscopy (NIRS) to assess some properties of a sub-arctic ecosystem. Basic Appl. Ecol. 7, 167–187 10.1016/j.baae.2005.05.002

[B58] Street-Perrott A. F. Barker P. A. (2008). Biogenic silica: a neglected component of the coupled global continental biogeochemical cycles of carbon and silicon. Earth Surf. Proc. Land. 33, 1436–1457 10.1002/esp.1712

[B59] Struyf E. Conley D. J. (2012). Emerging understanding of the ecosystem silica filter. Biogeochemistry 107, 9–18 10.1007/s10533-011-9590-2

[B60] Sundue M. (2009). Silica bodies and their systematic implications in Pteridaceae (Pteridophyta). Bot. J. Linn. Soc. 161, 422–435 10.1111/j.1095-8339.2009.01012.x

[B61] Tréguer P. J. De La Rocha C. L. (2013). The world ocean silica cycle. Annu. Rev. Mar. Sci. 5, 1–25 10.1146/annurev-marine-121211-172346 22809182

[B62] Vandevenne F. Struyf E. Clymans W. Meire P. (2012). Agricultural silica harvest: Have humans created a new and important loop in the global silica cycle? Front. Ecol. Environ. 10, 243–248 10.1890/110046

[B63] Van Soest P. J. Jones L. H. P. (1968). Effect of silica in forages upon digestibility. J. Diary Sci. 51, 1644–1648 10.3168/jds.S0022-0302(68)87246-7

[B64] Zhang C. Wang L. Zhang W. Zhang F. (2013). Do lignification and silicification of the cell wall precede silicon deposition in the silica cell of the rice (Oryza sativa L.) leaf epidermis? Plant Soil 372, 137–149 10.1007/s11104-013-1723-z

